# Parkinson’s Disease in Teneurin Transmembrane Protein 4 (*TENM4*) Mutation Carriers

**DOI:** 10.3389/fgene.2020.598064

**Published:** 2020-12-22

**Authors:** Jia-Li Pu, Ting Gao, Xiao-Li Si, Ran Zheng, Chong-Yao Jin, Yang Ruan, Yi Fang, Ying Chen, Zhe Song, Xin-Zhen Yin, Ya-Ping Yan, Jun Tian, Bao-Rong Zhang

**Affiliations:** Department of Neurology, College of Medicine, Second Affiliated Hospital, Zhejiang University, Hangzhou, China

**Keywords:** variant, genetic testing, Parkinson’s disease, pedigree, *TENM4*

## Abstract

**Introduction:**

Mutations in the teneurin transmembrane protein 4 (*TENM4*) gene, known to be involved in neuropsychiatric disorders, have been identified in three pedigree of essential tremor (ET) from Spain. ET has overlapping clinical manifestations and epidemiological symptoms with Parkinson’s disease (PD), suggesting these two disorders may reflect common genetic risk factors. In this study, we investigated clinical and genetic manifestations in four unrelated pedigrees with both ET and PD in which *TENM4* variants were identified.

**Methods:**

We subsequently explored whether *TENM4* variants contributed to the risk of developing PD. The frequency of *TENM4* variants was evaluated from four PD pedigrees and other 407 subjects.

**Results:**

The results revealed 12 different novel heterozygous variants, all at low frequency. A clear general enrichment of *TENM4* variants was detected in early onset PD patients (*p* < 0.001, OR = 5.264, 95% CI = 1.957–14.158).

**Conclusion:**

The results indicate that rare *TENM4* variants may be associated with an increased risk of PD.

## Introduction

Parkinson’s disease (PD), one of the most frequent neurodegenerative disorders, is mainly characterized by bradykinesia, resting tremor and rigidity (Lees et al., 2009). Interactions between environmental and genetic factors underlie the degeneration of nigral dopaminergic (DA) neurons and ensuing PD. Genetic factors account for ∼5−10% of PD cases (Deng et al., 2018). To date, 27 Mendelian genes have been reported to be linked with PD, and genome-wide association studies have succeeded in identifying many low-risk variants (Deng et al., 2018; Lunati et al., 2018).

Essential tremor (ET) is a common hyperkinetic movement disorder with an estimated prevalence of 5% among people over 65 years old (Deuschl et al., 2015). ET is characterized mainly by rhythmic, involuntary shaking of parts of the body, and occurs exclusively during voluntary movements or in positions against gravity. While the majority of PD cases are sporadic, ET has a strong genetic component, and more than half of affected individuals have a positive family history (Louis and Ottman, 2006). Although ET and PD are generally considered distinct entities, Spanaki and Plaitakis (2009) found ET occurred more frequently in relatives of PD patients, compared with that in controls. Furthermore, the risk of developing PD is up to fourfold greater in ET sufferers (Algarni and Fasano, 2018). The overlapping clinical manifestations and epidemiological symptoms suggest that PD and ET may underlie common genetic risk factors.

Mutations in the Teneurin Transmembrane Protein 4 (*TENM4*; MIM 610084) gene, known to be involved in neuropsychiatric disorders (Xue et al., 2018). have been identified recently in three pedigrees of ET from Spain (Hor et al., 2015). Additionally, *in vitro* and model organism analyses showed that mutations in *TENM4* gene result in protein mislocalization and axon guidance defects (Hor et al., 2015). However, further screening in a cohort of 269 Canadian ET cases and 288 matched controls revealed a negative association between *TENM4* and the Canadian population (Houle et al., 2017). In our previous study, Yan et al. (2020) found no evidence support that *TENM4* associated with ET. In addition, Chao et al. (2016) found that the c.4324 G > A mutation in *TENM4* originally identified by Hor and colleagues (Hor et al., 2015) was also present in the control group (379 ET cases and 398 healthy controls) in a Chinese population. Thus, similar studies have yielded inconsistent results.

Increasing evidence suggests that ET and PD may share genetic mutations, and a subset of patients may have a combination of long-standing ET with subsequent PD (ET-PD). Furthermore, one family with five affected individuals presented with either ET or PD, consistent with mutation of the of *PRKN* (*PARK2*) gene (Pellecchia et al., 2007). Unal Gulsuner et al. (2014) reported that High Temperature Requirement Protein A2 (*HTRA2*) is responsible for hereditary essential tremor and that homozygotes for this allele develop Parkinson disease. And Fused in sarcoma (*FUS*) mutations have been found in individuals with ALS/PD (Yan et al., 2010).

The aim of the present study was to further explore the associations between *TENM4* mutations and PD, and investigate whether *TENM4* variant carriers are at increased risk of developing PD. We first explored the clinical features and genetic features of four ET-PD pedigrees, then investigated whether *TENM4* variants might be associated with PD by comparing mutations in a cohort of sporadic PD cases and controls.

## Materials and Methods

### Family Study

#### Pedigrees

Four pedigrees of ET-PD with *TENM4* mutations were included in this study. Four probands and their family members were examined by two neurologists and genetically tested for neurodegenerative disorders (PD, ET, Alzheimer’s disease, etc.). Clinical and demographic features of the probands in four family pedigrees are described in “Results” section (Table 1). The study was approved by the Medical Ethics Committee of the Second Affiliated Hospital of Zhejiang University School of Medicine in accordance with the Declaration of Helsinki. All subjects participated in this study completed informed consent before the evaluation and original sample collection.

**TABLE 1:** Clinical manifestation of the probands in four family pedigrees.

Variants		Sex	AAO	IS	B	R	T	PI	Levodopa responsive	DK	DM
c.974G > A	p.R325Q	M	72	T	+	+	+	−	Responsive	−	−
c.2287C > T	p.R763C	F	52	B	+	+	−	−	Slightly good	−	−
c.6209G > A	p.R2070H	M	50	T	+	+	+	−	Not treated	−	−
c.5545A > G	p.T1849A	M	47	B	+	+	−	−	Responsive	−	−

*AAO, age at onset; IS, initial symptoms; B, bradykinesia; R, rigidity; T, resting tremor; PI, postural instability; DK, dyskinesia; DM, dementia; +, positive; −, negative.*

#### Genetic Analysis

Blood samples (2 ml) were collected from all cases, and genomic DNA was extracted from peripheral blood leucocytes using standard procedures. Probands and family members were screened for *TENM4* (NCBI transcript NM_001098816.2), *HTRA2* (NCBI transcript NM_013247), and *FUS* (NCBI transcript NM_004960.3) mutations by standard bi-directional Sanger sequencing of all coding exons and exon-intron boundaries (primer sequences available on request). Dosage analysis for *TENM4* exonic deletions and duplications was performed by multiplex ligation-dependent probe amplification (MLPA, MRC) (Mencacci et al., 2014). The other known PD pathogenic genes (*SNCA*, *GBA*, *LRRK2*, *UCHL1*, *VPS35*, *PRKN*, *PINK1*, *DJ-1*, *ATP13A2*, *GIGYF2*, *PLA2G6*, *HtrA2*, *FBXO7*, *SYNJ1*, *DNAJC6*, *DNAJC13*, *CHCHD2*, *Rab39B*) were also analyzed in all participants.

### Target Sequencing, Variant Filtering, Identification, and Analysis

#### Participants

The study included 207 unrelated patients with PD and 200 healthy control subjects from East China. Healthy controls were recruited from local communities. All patients were enrolled from outpatient neurology clinics of the Second Affiliated Hospital of Zhejiang University School of Medicine and local communities, and evaluated by two movement disorder specialists for diagnosis of PD according to criteria provided by the Movement Disorder Society (Postuma et al., 2015). The exclusion criteria were described in our previous study (Gao et al., 2019). To summarize, participants with secondary causes of parkinsonism such as vascular, drug-induced, and toxin-induced, and other neurodegenerative diseases such as progressive supranuclear palsy, multiple system atrophy, essential tremor, Wilson’s disease and ET convert to PD were excluded. Additionally, other internal diseases which might also present tremor symptom such as hyperthyroidism were also excluded. The protocol was approved by the Medical Ethics Committee of the Second Affiliated Hospital of Zhejiang University School of Medicine in accordance with the Declaration of Helsinki. Written informed consent was completed for every participant before the evaluation and sample collection.

#### DNA Preparation, Target Resequencing, Variant Filtering, Validation, and Analysis

*TENM4* and two additional ET-related genes (*HTRA2* and *FUS*) were selected as targeted genes for capturing and sequence analyses. Molecular inversion probes (MIPs) were designed to capture all exons and intron-exon boundaries (5 bp flanking sequences) of target genes (Yang et al., 2019). Briefly, fragmented genomic DNA was captured by a customized array designed to target all exons, splicing sites, and flanking intronic sequences of the three genes (NimbleGen, Roche). Captured DNA fragments were sequenced on an Illumina HiSeq2000 Analyzer (Ahmed et al., 2003). Variants were filtered based on a read depth ≥ 4×, a genotype quality ≥ 20, and the proportions of reads with alternative alleles ≥ 0.3. Two publicly available resources were used to obtain variant frequency data; the 1000 Genomes Project and the Exome Aggregation Consortium (Dec 2019).

#### Criteria for Pathogenicity of Rare Variants

All non-synonymous variants were analyzed by a database of human non-synonymous SNVs and their functional predictions and annotations (dbNSFP, versions 3.3–3.5) (Liu et al., 2016). For interpretation of the validated variants, multiple prediction indices were adopted to clarify their pathogenicity, and variants were considered as likely pathogenic based on Sorting Intolerant From Tolerant (SIFT) score < 0.05 (Ng and Henikoff, 2003), Polyphen-2 score > 0.86 (Adzhubei et al., 2013) and Combined Annotation-Dependent Depletion (CADD) score > 12.35 (Kircher et al., 2014).

#### Statistical Analysis

Variants with a minor allele frequency < 0.1% (gnome AD or 1,000 G) were defined as rare variants and included in the gene-based burden test. The association between rare variants and PD was analyzed using Fisher’s exact tests, odds ratio (OR) and 95% confidence intervals (CI). All statistical analyses were performed using IBM SPSS Statistics 23.0^1^, and two-tailed *p* < 0.05 was considered statistically significant.

## Results

### Family Study

#### Family A

**FIGURE 1:**
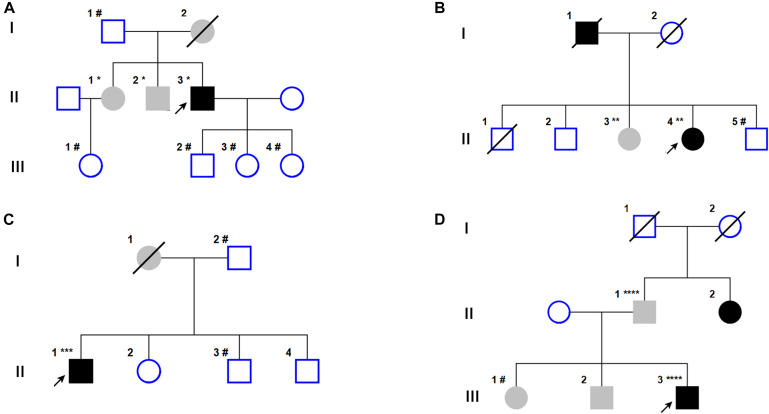
Pedigrees of the four families with TENM4 mutations involved in this study. **(A–D)** Pedigress of four different families with TENM4 mutations. Notes: black symbols, individuals with ET and PD; blank symbols, unaffected; gray symbols, individuals who are reported to have ET by history but some are not examined; arrow, probands; diagonal lines, deceased individuals; circle, female; square, male. ^∗^: c.974 G > A/wild type; ^∗∗^: c.2287 C > T/wild type; ^∗∗∗^: c.6209 G > A/wild type; ^*⁣*⁣**^: c.5545 A > G/wild type; ^#^: wild type/wild type.

The proband (Case II-3, Figure 1A) was a 75 year-old right-handed male of East Chinese origin with PD, with disease onset at age 72, and tremor of the left foot and arm. He now presents with anosmia, constipation, progressive loss of dexterity and slowness in the left foot. Examination showed an asymmetric rigid-akinetic parkinsonian syndrome with rest tremor and bradykinesia in the left foot and arm. Postural instability, dyskinesia and dementia were not observed. The efficacy of levodopa therapy was responsive and symptoms slightly relieved. His mother (Case I-2) passed away but was described with tremor in both hands. Whereas, further clinical information couldn’t be acquired. Examination of the proband’s brother and sister revealed kinetic tremor in both hands, without dyskinesia or hypertonia. They were clinically diagnosed of essential tremor and on no medications regards of mild symptoms.

Gene screening in this family revealed one variant, *TENM4* c.974G > A; p.R325Q (carried by Case II-3, II-1, and II-2), in the proband and his sister and brother. The R325Q (rs373911172) mutation has not been reported previously in ET or PD. Children of the proband and that of his sister were unaffected and non-carriers, as well as father of the proband. No rare variants of *HTRA2* or *FUS* were detected in any of the tested family members. *PRKN* and *LRRK2* gene mutations were found in the proband, while the pathogenicity analysis assigned the mutations as benign.

#### Family B

The proband (Case II-4, Figure 1B) was a 55 year-old female with bradykinesia, rest tremor in lower limbs and being first diagnosed with PD 6 years ago. She complained of bradykinesia and poor dexterity, and suffered tremor in both hands 1 year ago. The symptoms slightly ameliorated after taking levodopa. Her father (Case I-1) was diagnosed with PD in his sixties and died 5 years ago. Her sister (Case II-3) presented with head tremors when nervous or excited, while bradykinesia and rest tremor were not observed. Genetic tests revealed that the proband and her sister carried *TENM4* c.2287C > T; p.R763C. One of her brothers (Case II-5) was not a carrier. Rare variants of *HTRA2* and *FUS* were not identified. Genetic analysis data were not available for other family members. No other PD pathogenic gene mutations were found in the proband.

#### Family C

The proband (Case II-1, Figure 1C) was a 72 year-old male who presented with bilateral hand tremors at the age of 50. He recently attended the outpatient clinic for 2 years for stiffness of facial expression and slowness of the left hand and foot. No dopaminergic drugs had been prescribed. His mother (Case I-1) had a history of tremor in both hands, but did not experience dexterity or walking problems. No tremor or bradykinesia were observed in his brothers or sister.

The proband was heterozygous for *TENM4* c.6209G > A; p.R2070H. *LRRK2* mutation was found in the proband and allocated as benign by rare variant pathogenicity analysis. No rare variants were detected for *HTRA2* or *FUS*. His father and brother (Case II-3) are non-carriers for *TENM4* c.6209G > A. Unfortunately, genetic information for other members of the family was unavailable.

#### Family D

The proband (Case III-3, Figure 1D) was a 51 year-old right-handed male with bradykinesia been diagnosed as Parkinson’s disease for 4 years. Physical examination showed a mask face and rigid-akinetic parkinsonian syndrome without tremor of the right lower extremities. Dopaminergic therapy resulted in tangible improvement in parkinsonian symptoms. 11C-labeled 2β-carbomethoxy-3β-(4-fluorophenyl) tropane positron emission tomography/computed tomography (11^*C*^-CFT PET/CT) analysis revealed an asymmetric bilateral reduced tracer uptake, more marked in the left putamen. His sister (Case III-1), brother (Case III-2), and father (Case II-1) presented with tremor of both hands but without bradykinesia and rigidity. By contrast, his aunt had difficulty walking and poor dexterity and was diagnosed as Parkinson’s disease in her sixties.

Gene sequencing of all available family members revealed that the proband and his father were heterozygous for the rare *TENM4* c.5545A > G; p.T1849A mutation, while his sister (Case III-1) was a non-carrier. *PRKN* and *LRRK2* mutations were found in the proband, however, rare variant pathogenicity analysis determined them as benign mutations.

### Targeted Gene Panel Sequencing

We hypothesized that pathogenic variants in *TENM4* may also be found in subjects with PD without a family history. To investigate this, we examined targeted gene panel sequencing data for a large cohort of patients predominantly affected by PD, alongside controls. In total, 207 patients with sporadic PD (male/female = 112/95, age = 52.83 ± 10.56 years) and 200 healthy control participants (male/female = 85/115, age = 46.29 ± 11.05 years) were included and analyzed (Table 2). The percentage read depth of target genes was 98, 96, and 92% of bases covered by at least 4×, 10×, and 20×, respectively.

**TABLE 2:** Summary of sporadic PD-control demographics.

Series	Number	Age	Male/Female
Total PD	207	52.83 ± 10.56	112/95
EOPD (AAO ≤ 50)	100	44.74 ± 7.34	53/47
LOPD (AAO > 50)	107	60.34 ± 6.86	59/48
Controls	200	46.29 ± 11.05	85/115

*PD, Parkinson’s disease; EOPD, early onset Parkinson’s disease; LOPD, late onset Parkinson’s disease; AAO, age at onset.*

**FIGURE 2:**
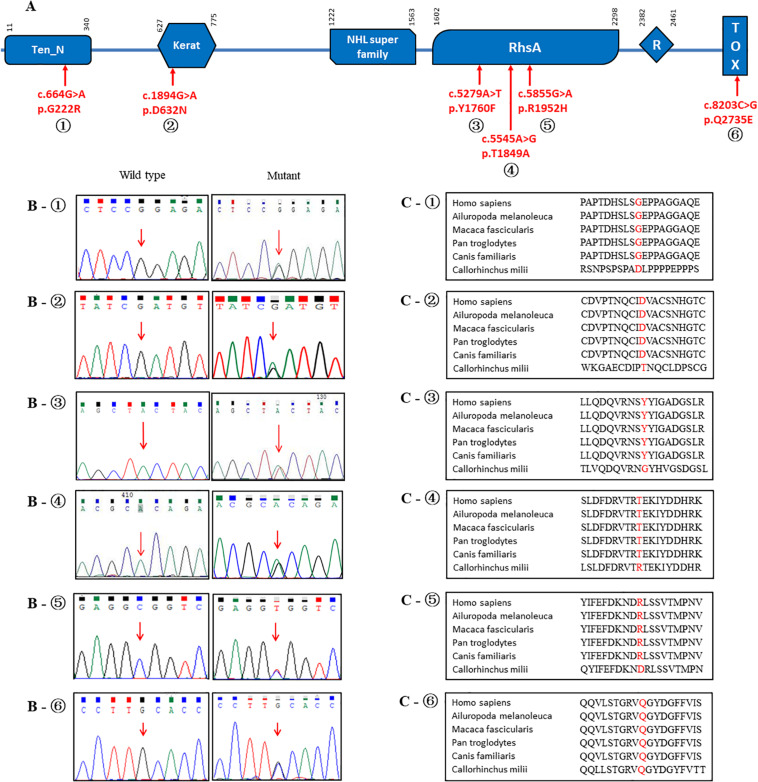
Schematic representations of the *TENM4* protein with blue boxes indicating different domains. **(A)** Variants found in present study are indicated by arrows. **(B)** Electropherograms of the sequence of the variants. **(C)** The position and surroundings of those variants are highly conserved across different species.

Overall, 12 rare non-synonymous-coding variants with a minor allele frequency < 0.1% were identified in the exon regions of the *TENM4* gene after applying quality filter (Table 3). None of these rare variants have been reported previously, and all were categorized as disease-causing based on SIFT, Polyphen-2 and CADD values, and remained conserved based on GERP + + prediction (Ioannidis et al., 2016). The structures and functions were predicted as altered structures and/or functions (Supplementary Materials). Unfortunately, due to technical issues, one of the variants (p.R763C) could not be sequenced in healthy controls. In addition, six of these variants (p.R1952H, p.T1849A, p.Y1760F, p.D632N, p.G222R, and p.Q2735E) were absent in our gender-matched healthy control cohort (Table 3), and their locations are depicted in Figure 2. However, no rare variants of *HTRA2* or *FUS* were detected in PD or healthy controls. None of the previously reported PD risk genes had been identified in all participants.

**TABLE 3:** Summary of variants of TENM4 classified as likely pathogenic in the cohort.

Variants	Position	Freq. patient (%)	Freq. control (%)	Freq. 1,000 G	Freq. ExAC	dbSNP ID	SIFT score	Polyphen score	CADD score	GERP++ score
p.R763C	78498021	0.4651	NA	NA	1.03E-4	rs751467112	0	0.998	35	4.98
p.R325Q	78600940	0.9302	0.5000	3.99E-4	4.60E-5	rs373911172	0.009	0.999	35	4.81
p.R1952H	78381535	0.4651	0	3.99E-4	2.09E-4	rs140341040	0.205	1	24.8	4.93
p.T1849A	78383326	0.9302	0	NA	1.08E-4	rs772977333	0.003	0.994	24.6	5.65
p.Y1760F	78387414	0.9302	0	NA	8.72E-6	rs745395614	0.01	0.997	24.3	4.71
p.R2733Q	78369215	0.4651	2.0000	7.99E-4	8.34E-6	rs185503085	0.047	0.85	23.2	5.65
p.D632N	78523251	0.9302	0	3.99E-4	2.82E-4	rs370767956	0.221	0.923	23.2	4.94
p.L1937V	78381581	0.4651	1.5000	7.99E-4	NA	rs192931562	0.238	0.997	22.8	4.81
p.V2423M	78380123	0.4651	0.5000	NA	8.34E-6	rs759903805	0.024	0.999	26.5	5.67
p.A1165T	78437181	0.4651	1.0000	2.00E-4	4.14E-5	rs550968777	0.121	0.968	23.9	5.32
p.G222R	78614398	0.4651	0	3.99E-4	2.20E-4	rs541146378	0.076	0.989	23.5	5.64
p.Q2735E	78369210	0.4651	0	NA	8.28E-6	rs527857070	0.242	0.969	12.59	5.65

*Threshold values for deleteriousness: SIFT-less than 0.05; Polyphen-2 greater than 0.86; CADD-greater than 12.35; GERP++ is a score for the conservation of the amino acid; scores > 3 can be considered as highly conserved. NA, not found; Freq, frequency; 1,000 G, 1,000 Genomes Project; ExAC, Exome Aggregation Consortium.*

### Gene-Based Burden Analysis

To determine whether these rare variants of *TENM4* contribute collectively to sporadic PD risk in our cohort, we performed a gene-based burden analysis using Fisher’s exact test (Nicolae, 2016), and a clear general enrichment was detected for early onset Parkinson’s disease (EOPD, age at onset ≤ 50) patients (*p* < 0.001, OR = 5.264, 95% CI = 1.957–14.158). No significant differences were found for late onset Parkinson’s disease (LOPD, age at onset > 50) or total PD (Supplementary Table 1).

## Discussion

Advances in next-generation sequencing have revealed a growing number of causal genes in Mendelian form PD patients (Deng et al., 2018). However, a large number of early onset cases still remain to be explained, which indicates that there are many genetic factors yet to be clarified. The connection between PD and ET has received much attention (Jankovic, 1989). Accumulating evidence supports an association between PD and ET, including overlapping clinical features, obviously increased prevalence of PD in patients with a family history of ET, and increased prevalence of ET in family members of PD patients (Tarakad and Jankovic, 2018).

Recent discoveries confirmed links between ET and *LINGO1*, *FUS*, and *TENM4* (Hor et al., 2015; Clark and Louis, 2018). In our current family study, we assessed four unrelated ET and PD pedigrees (family coexistence of ET and PD) in which *TENM4* variants were identified in individuals without evidence of mutations in *LINGO1* or *FUS* genes. Most cases presenting PD phenotypes were *TENM4* variant carriers. Thus, we speculated that *TENM4* may be linked to the risk of developing PD.

We subsequently identified 12 novel rare variants of *TENM4* in a Chinese cohort of sporadic PD patients that may be associated with PD developing, including five that were also present in controls. With other PD related genes tested in our sporadic PD patients, no significant risk genes were found, which therefore strengthen our hypothesis that mutations in TNEM4 gene may associated with PD. Burden analysis indicated no overrepresentation of variant alleles in sporadic PD cases, but did reveal an association between *TENM4* rare variants and disease in EOPD case-controls. It should be noted that the results of burden analysis can be impacted by the detection methods, read depth, and data from the GnomAD database (compared to using ethnically-, age-, and gender-matched controls). Thus, data from burden analysis should be interpreted cautiously. However, the results implied *de novo* variants or incomplete penetrance.

Despite dramatic advances in our understanding of the genetic basis of PD, a large number of early onset and sporadic cases still remain to be clarified. There is a possible functional link between Mendelian genes and sporadic PD, and previous studies suggest that rare and low-frequency variants of PD Mendelian genes may play a role in sporadic forms of the disease (Kun-Rodrigues et al., 2015; Spataro et al., 2015). In addition, previous research confirmed the positive contribution of rare coding GTP cyclohydrolase1 (*GCH1*), the causative gene in dopamine related dystonia (DRD) for which gene variants have been identified in a large cohort of sporadic PD cases (Mencacci et al., 2014). Our present study is the first to link ET with the *TENM4* gene in PD cases. Those PD patients with *TENM4* mutations mildly response to levodopa treatment in four pedigrees indicated an undefined mechanism of gene-related on dopaminergic therapy of PD.

The pathogenetic mechanism linking *TENM4* mutations with ET is uncertain. Biochemical evidence from *TENM4*-deficient mice revealed loss of embryonic mesoderm and differentiation in a cell-autonomous manner (Nakamura et al., 2013). Furthermore, functional studies are needed to elucidate the importance mutations in this gene.

The limited contribution of the *TENM4* gene to PD revealed by our study could be due to a lack of functional studies confirm pathogenic variants. Furthermore, we evaluated a cohort of sporadic PD cases in which *TENM4* variants may not reflect the frequency in familial cases. The relatively small sample size and absence of family co-segregation may be limitations of our study.

In conclusion, we provide evidence that rare *TENM4*-coding variants may be considered a risk factor for PD. However, determining how *TENM4* mutations known to cause ET may be related to risk alleles in PD requires further investigation. Due to racial heterogeneity and the limited sample size of our cohort, more robust independent studies are needed to further illuminate the relationship between PD and *TENM4* gene variants.

## Data Availability Statement

The raw data supporting the conclusions of this article will be made available by the authors, without undue reservation.

## Ethics Statement

The studies involving human participants were reviewed and approved by the Medical Ethics Committee of the Second Affiliated Hospital of Zhejiang University School of Medicine. The patients/participants provided their written informed consent to participate in this study. Written informed consent was obtained from the individual(s) for the publication of any potentially identifiable images or data included in this article.

## Author Contributions

J-LP and TG were involved with study concept and design, acquisition of data, analysis, and interpretation of data, drafting and revising the manuscript. TG, X-LS, RZ, C-YJ, YR, YF, and YC were involved with acquisition of data, analysis, and interpretation of data. B-RZ, J-LP, JT, ZS, Y-PY, and X-ZY were involved with PD patients’ recruitment. B-RZ and JT were involved with revising the manuscript and were responsible for supervision of study. All authors listed meet the criteria for authorship.

## Conflict of Interest

The authors declare that the research was conducted in the absence of any commercial or financial relationships that could be construed as a potential conflict of interest.

## Footnotes

1 https://www.ibm.com/support/knowledgecenter/SSLVMB_23.0.0/spss/product_landing
