# Supplementary material for: Parkinson’s Disease in Teneurin Transmembrane Protein 4 (TENM4) Mutation Carriers
Source: Front Genet. 2020 Dec 22;11:598064. doi: 10.3389/fgene.2020.598064 (PMC7783409; doi:10.3389/fgene.2020.598064)
Supplement: Supplementary file 1 [file Data_Sheet_1.docx]

# Supplementary materials

## Supplemental Table 1 Summary of the gene-based burden test

| *TENM4* | All rare variants involved | | | |
| --- | --- | --- | --- | --- |
|  | Cases | Controls/200 | *p*-value | OR (95% CI) |
| EOPD/100 | 14 | 6 | <0.001^a^ | 5.264 (1.957 - 14.158) |
| LOPD/107 | 2 | 3 | 1.000 | 1.251 (0.206 - 7.603) |
| Total PD/207 | 16 | 9^b^ | 0.175 | 1.779 (0.767 - 4.122) |

^a^ For statistically significant.

^b^ Variants of position 78498021, 78381535, 78383326, 78387414, 78523251, 78614398, 78369210 in *TENM4* were not found in controls.

Abbreviations: PD, Parkinson’s disease; EOPD, early onset Parkinson’s disease; LOPD, late onset Parkinson’s disease; AAO, age at onset; OR, odds ratio; CI, confidential interval.

**Supplementary Table 2** Summary of predicted functions of *TENM4* protein and 12 variants

| **Variants** | **Position** | **Biological Process Predictions** | **Probability** | **Molecular Function Predictions** | **Probability** | **Cellular Component Predictions** | **Probability** |
| --- | --- | --- | --- | --- | --- | --- | --- |
| ***TENM4*_p1** |  | **transport** | **0.799** | **catalytic activity** | **0.897** | **membrane** | **0.946** |
| p.G222R | 78614398 | transport | 0.797 | catalytic activity | 0.904 | membrane | 0.946 |
| p.R325Q^a^ | 78600940 | regulation of metabolic process | 0.804 | catalytic activity | 0.897 | membrane | 0.946 |
| p.D632N^a^ | 78523251 | regulation of metabolic process | 0.835 | catalytic activity | 0.857 | membrane | 0.947 |
| p.R763C^a^ | 78498021 | regulation of metabolic process | 0.813 | catalytic activity | 0.858 | membrane | 0.945 |
| p.A1165T^a^ | 78437181 | regulation of metabolic process | 0.802 | catalytic activity | 0.898 | membrane | 0.946 |
| ***TENM4*_p2** |  | **RNA splicing** | **0.966** | **cytoskeletal protein binding** | **0.906** | **membrane** | **0.650** |
| p.Y1760F^b^ | 78387414 | RNA splicing | 0.948 | cytoskeletal protein binding | 0.905 | nucleolus | 0.659 |
| p.T1849A^b^ | 78383326 | RNA splicing | 0.961 | cytoskeletal protein binding | 0.908 | nucleolus | 0.675 |
| p.L1937V^b^ | 78381581 | RNA splicing | 0.967 | cytoskeletal protein binding | 0.907 | nucleolus | 0.648 |
| p.R1952H^b^ | 78381535 | RNA splicing | 0.937 | cytoskeletal protein binding | 0.902 | nucleolus | 0.671 |
| p.V2423M | 78380123 | RNA splicing | 0.964 | cytoskeletal protein binding | 0.900 | membrane | 0.645 |
| p.R2733Q^b^ | 78369215 | RNA splicing | 0.967 | cytoskeletal protein binding | 0.908 | nucleolus | 0.665 |
| p.Q2735E | 78369210 | RNA splicing | 0.963 | cytoskeletal protein binding | 0.901 | membrane | 0.649 |

**^a^ for altered biological process predictions. ^b^ for altered cellular component predictions.**

**Supplementary Figure Legends**

**S_Figure 1** Structure of *TENM4* protein part 1 (1-1380 amino acids)

**S_Figure 2** Structure of *TENM4* protein part 2 (1381-2769 amino acids)

**S_Figure 3-14** Structures of parts of variants of *TENM4* (p.R763C, p.R325Q, p.R1952H, p.T1849A, p.Y1760F, p.R2733Q, p.D632N, p.L1937V, p.V2423M, p.A1165T, p.G222R and p.Q2735E). The mutated location is labeled in red box.
