# Supplementary figures and images for: Parkinson’s Disease in Teneurin Transmembrane Protein 4 (TENM4) Mutation Carriers
Source: Front Genet. 2020 Dec 22;11:598064. doi: 10.3389/fgene.2020.598064 (PMC7783409; doi:10.3389/fgene.2020.598064)

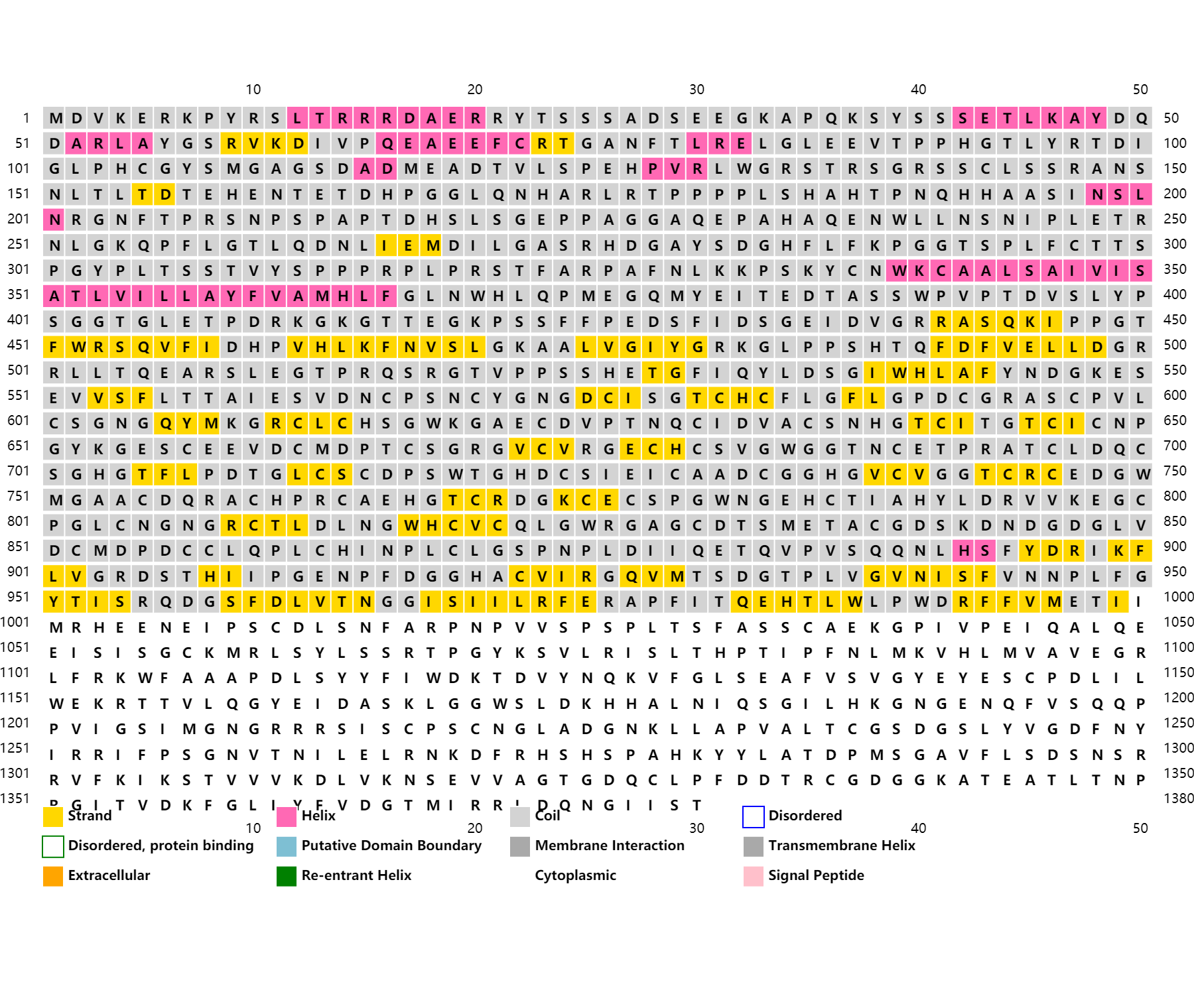

Supplement: Supplementary file 2 [file Image_1.PNG]

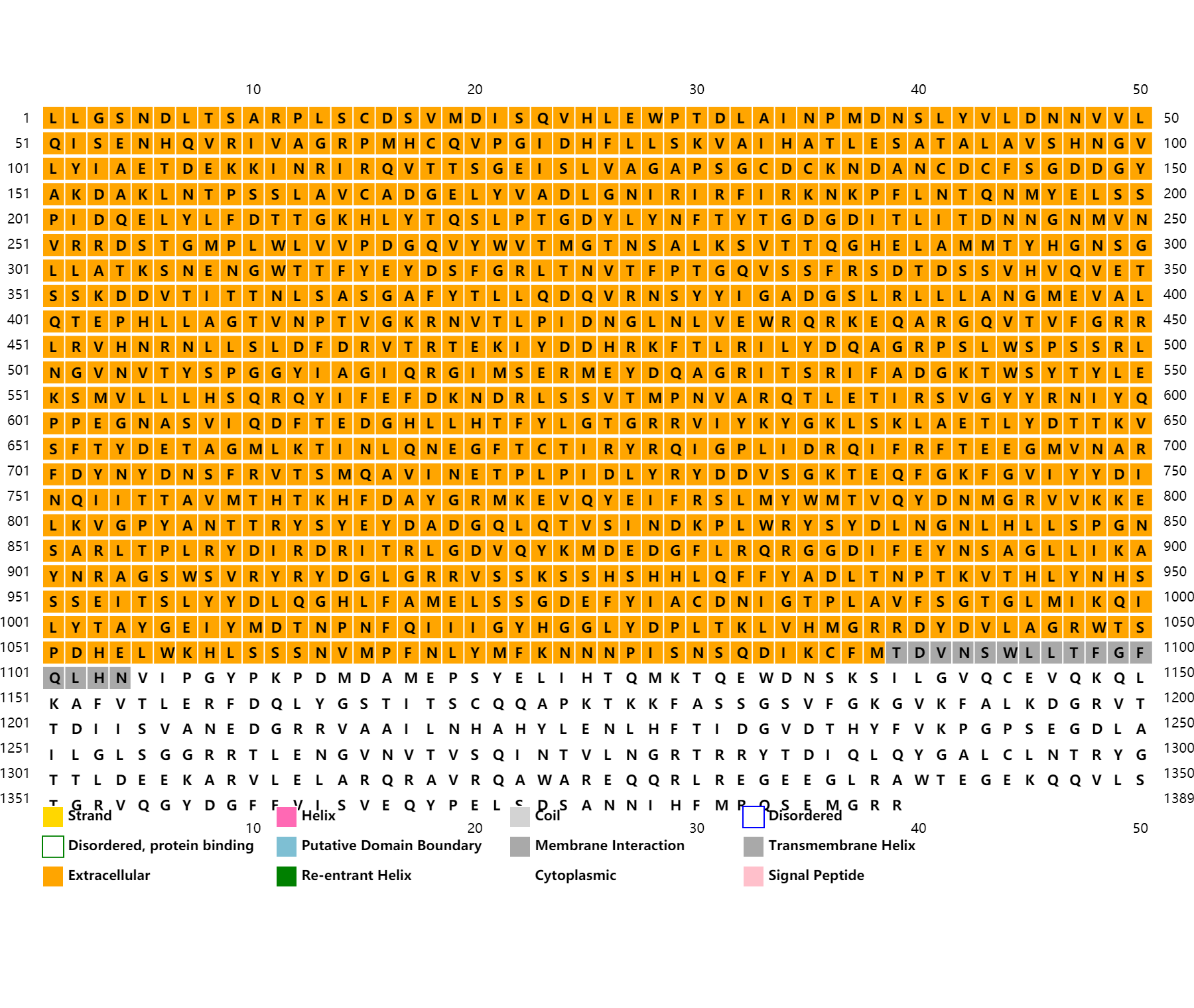

Supplement: Supplementary file 3 [file Image_2.PNG]

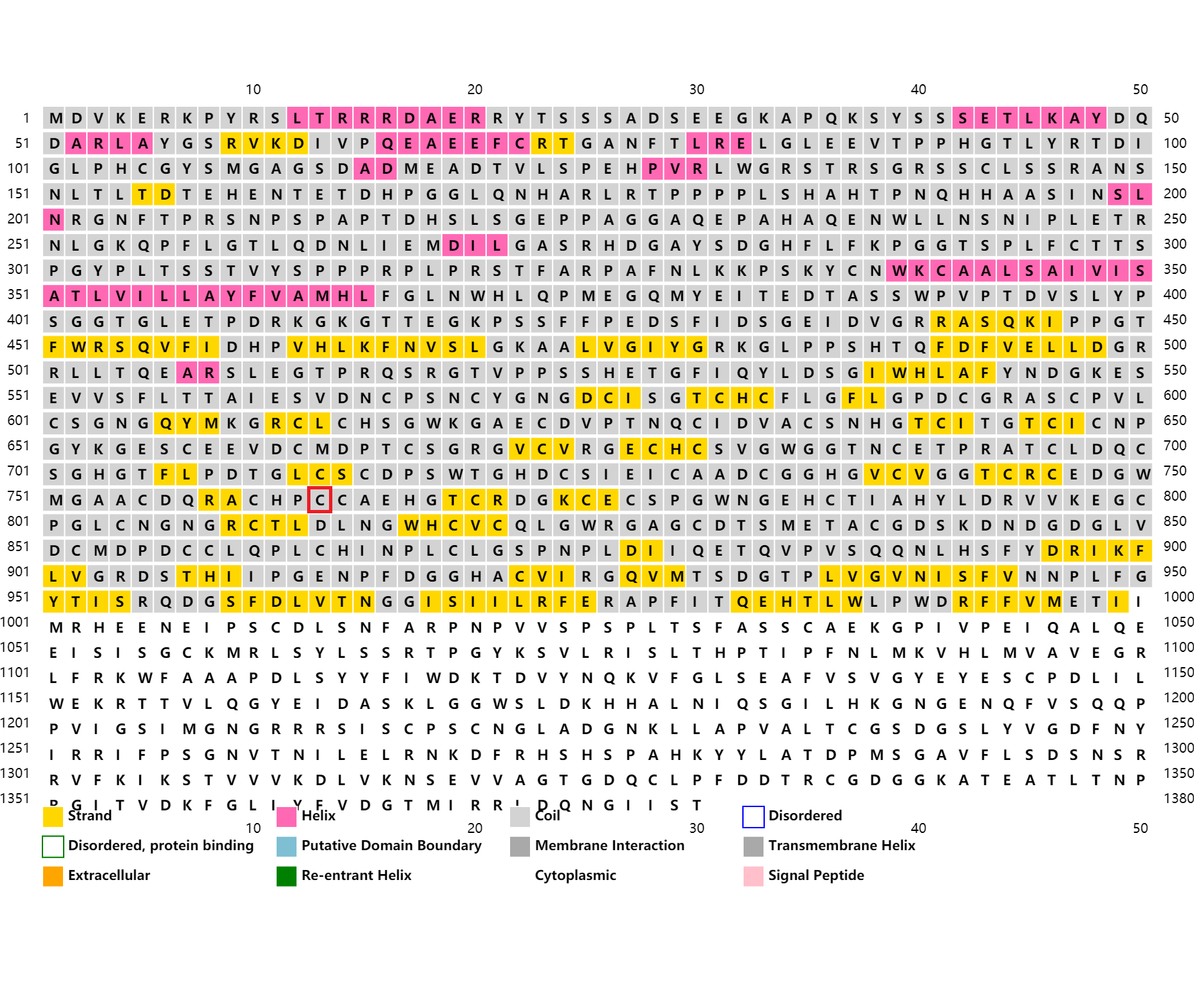

Supplement: Supplementary file 4 [file Image_3.PNG]

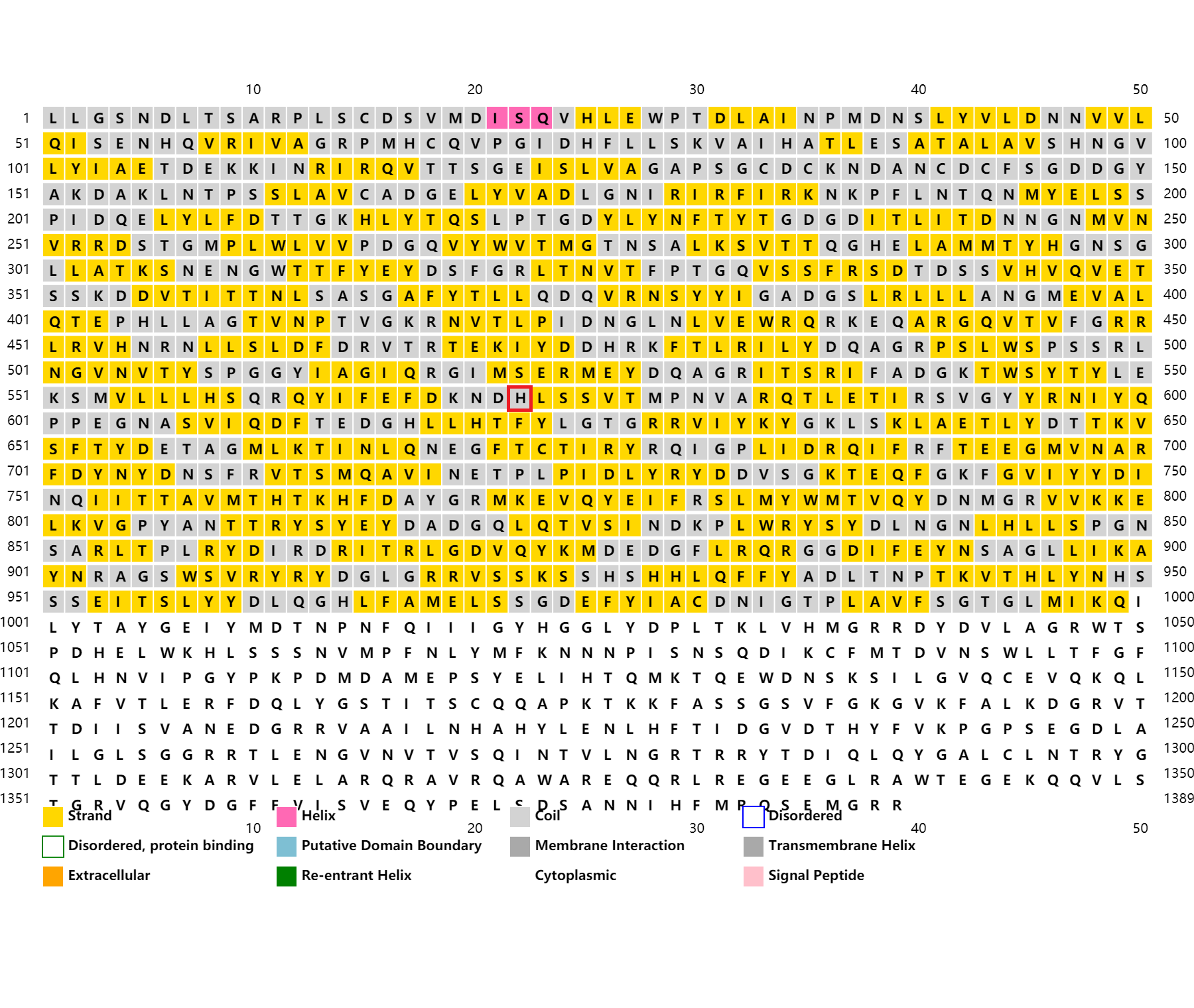

Supplement: Supplementary file 5 [file Image_5.PNG]

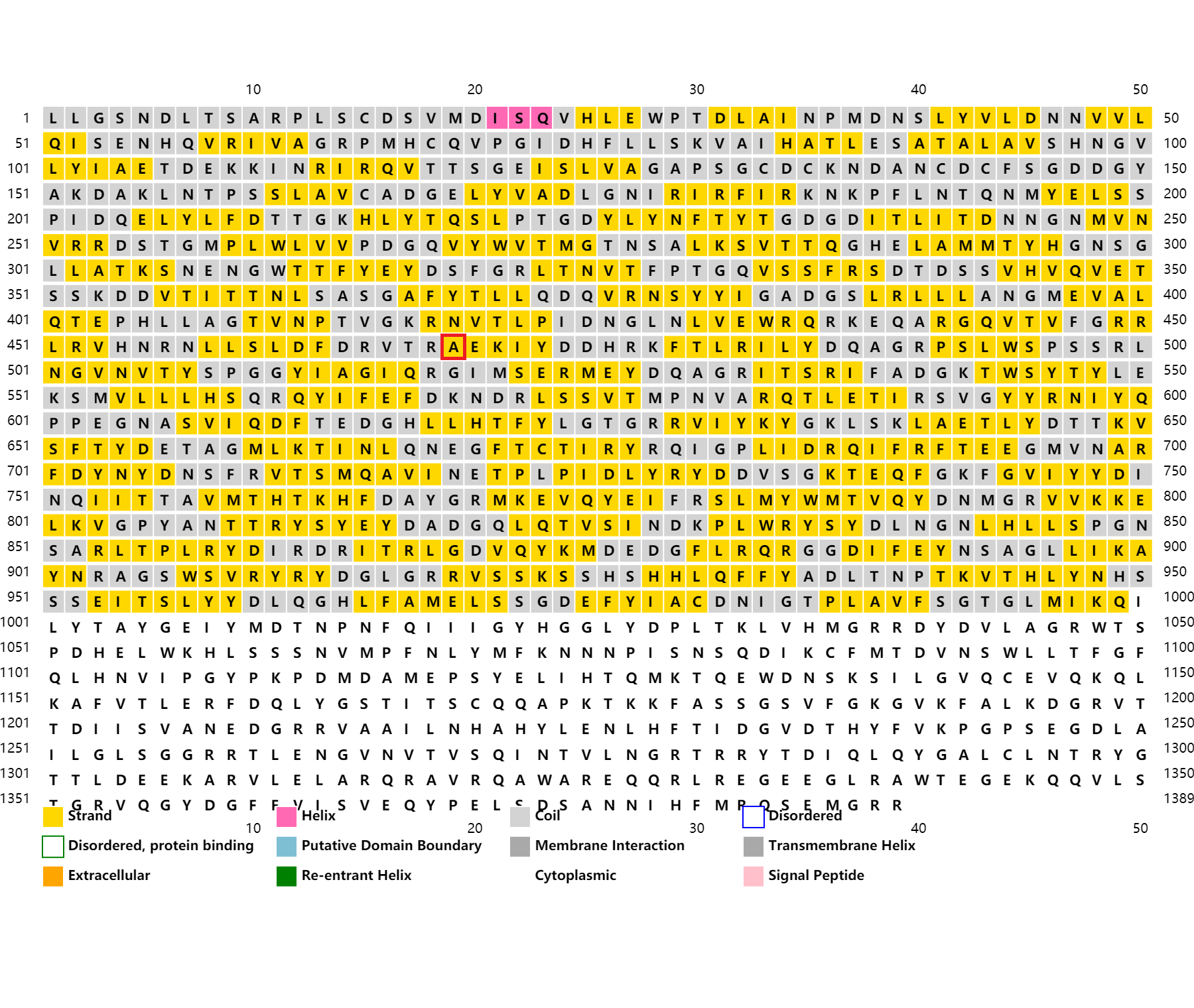

Supplement: Supplementary file 6 [file Image_6.PNG]

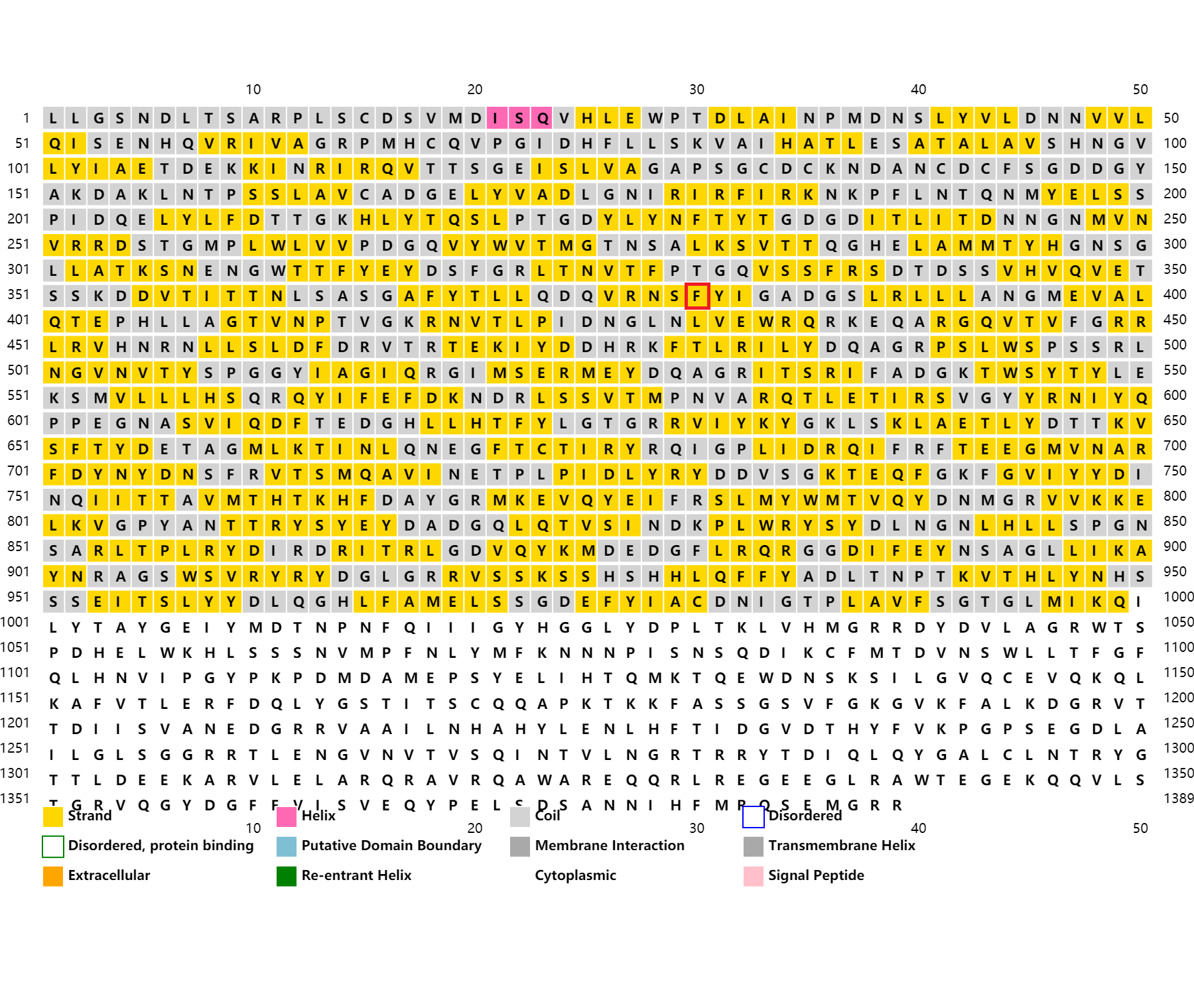

Supplement: Supplementary file 7 [file Image_7.PNG]

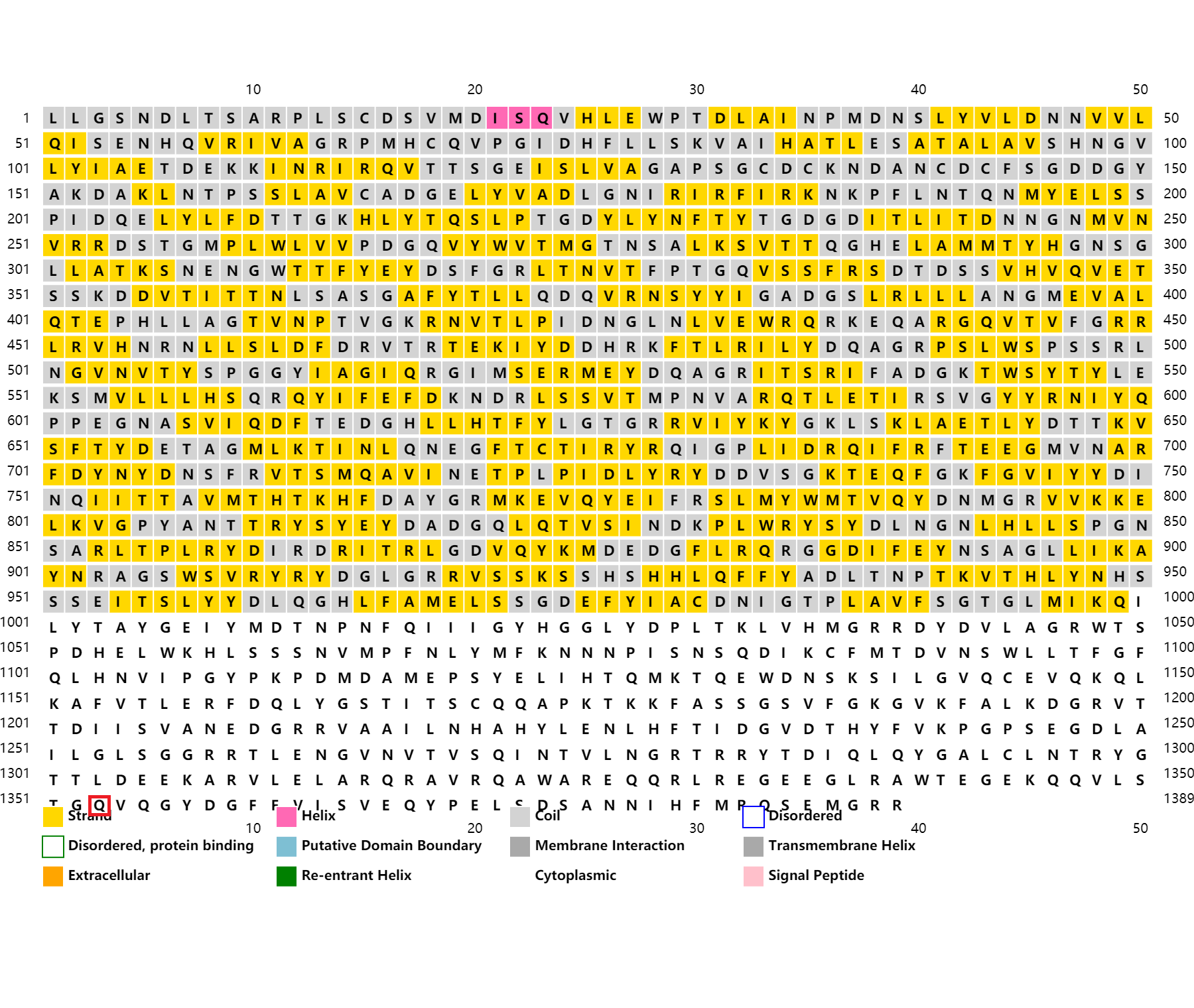

Supplement: Supplementary file 8 [file Image_8.PNG]

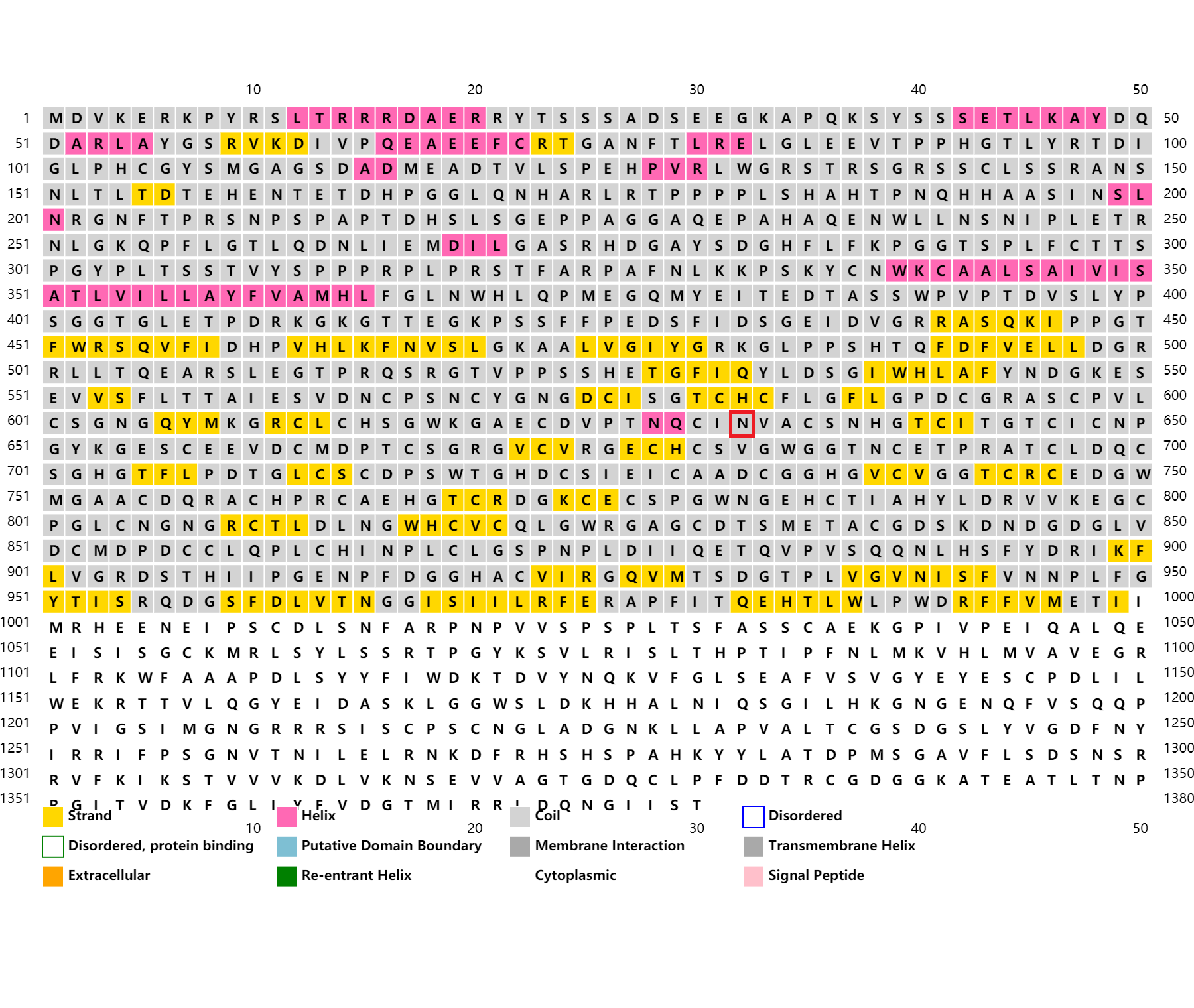

Supplement: Supplementary file 9 [file Image_9.PNG]

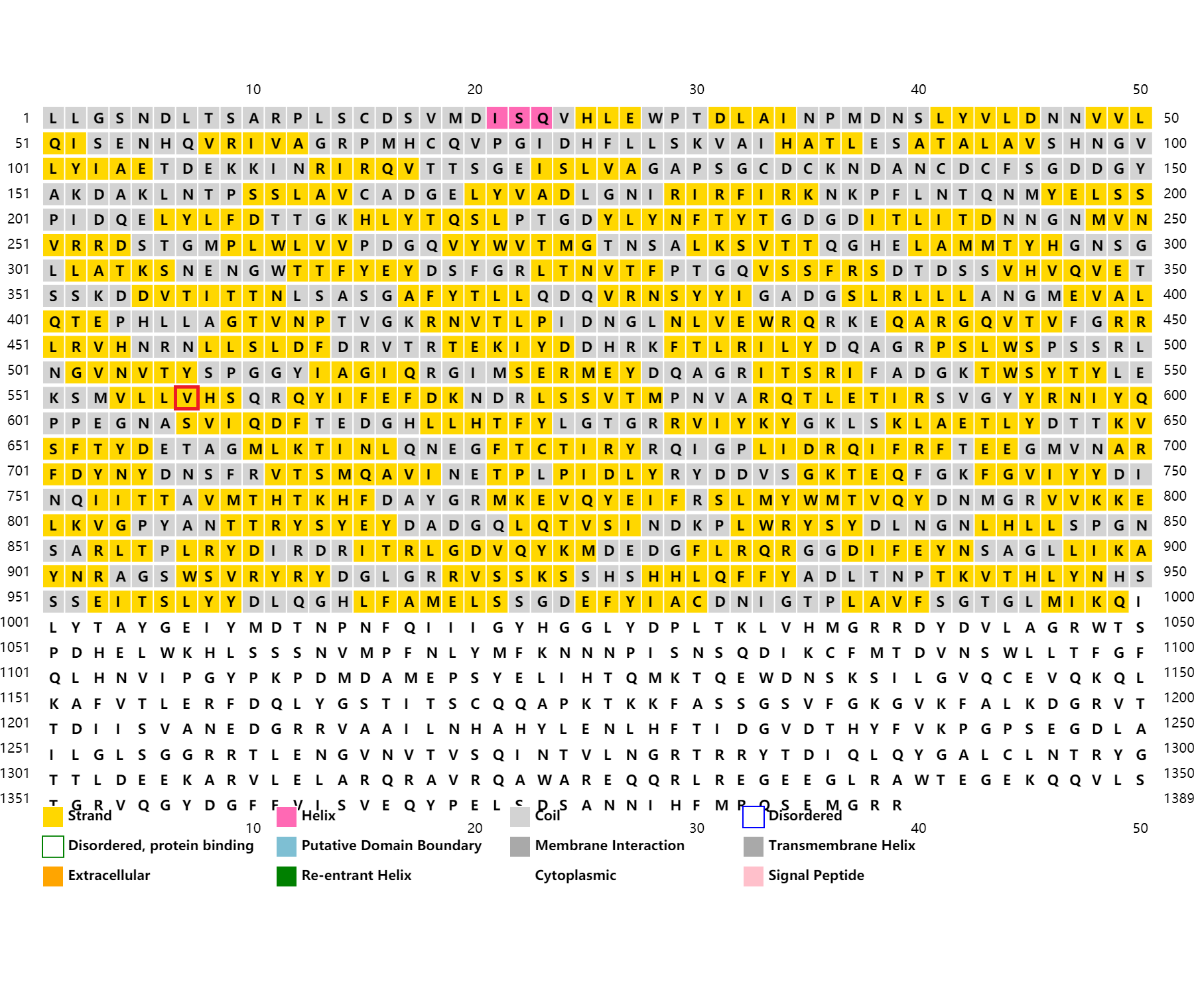

Supplement: Supplementary file 10 [file Image_10.PNG]

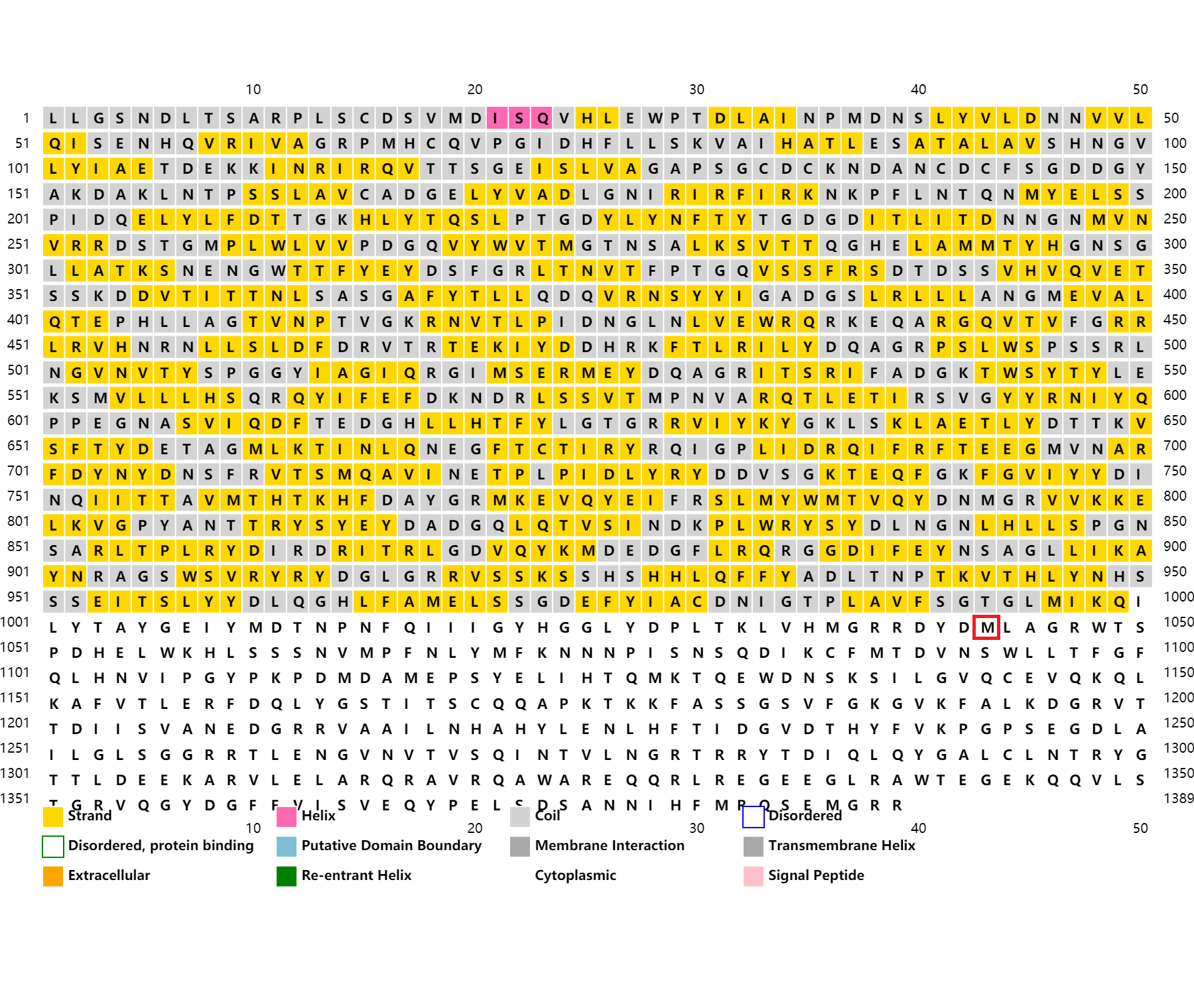

Supplement: Supplementary file 11 [file Image_11.PNG]

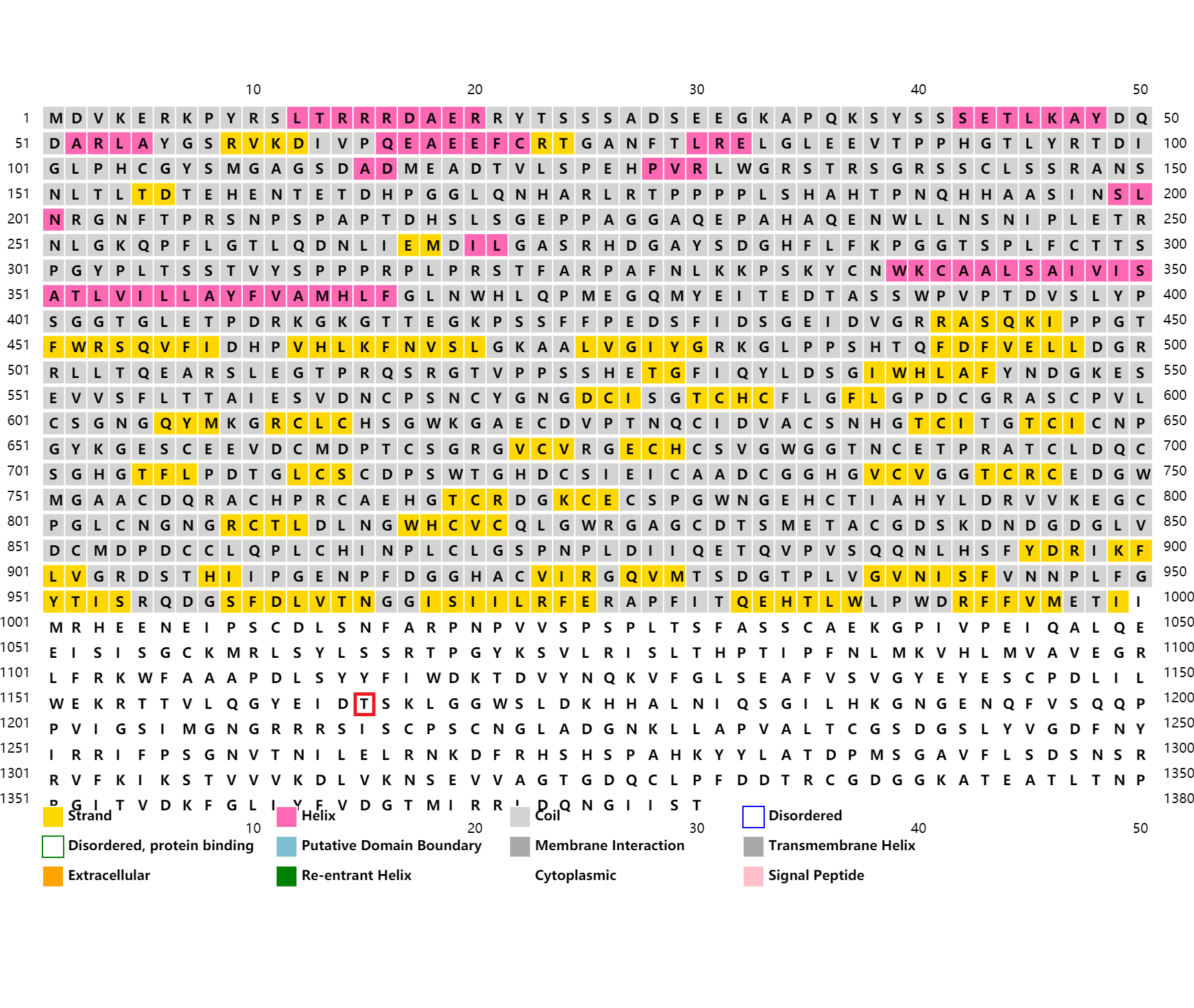

Supplement: Supplementary file 12 [file Image_12.PNG]

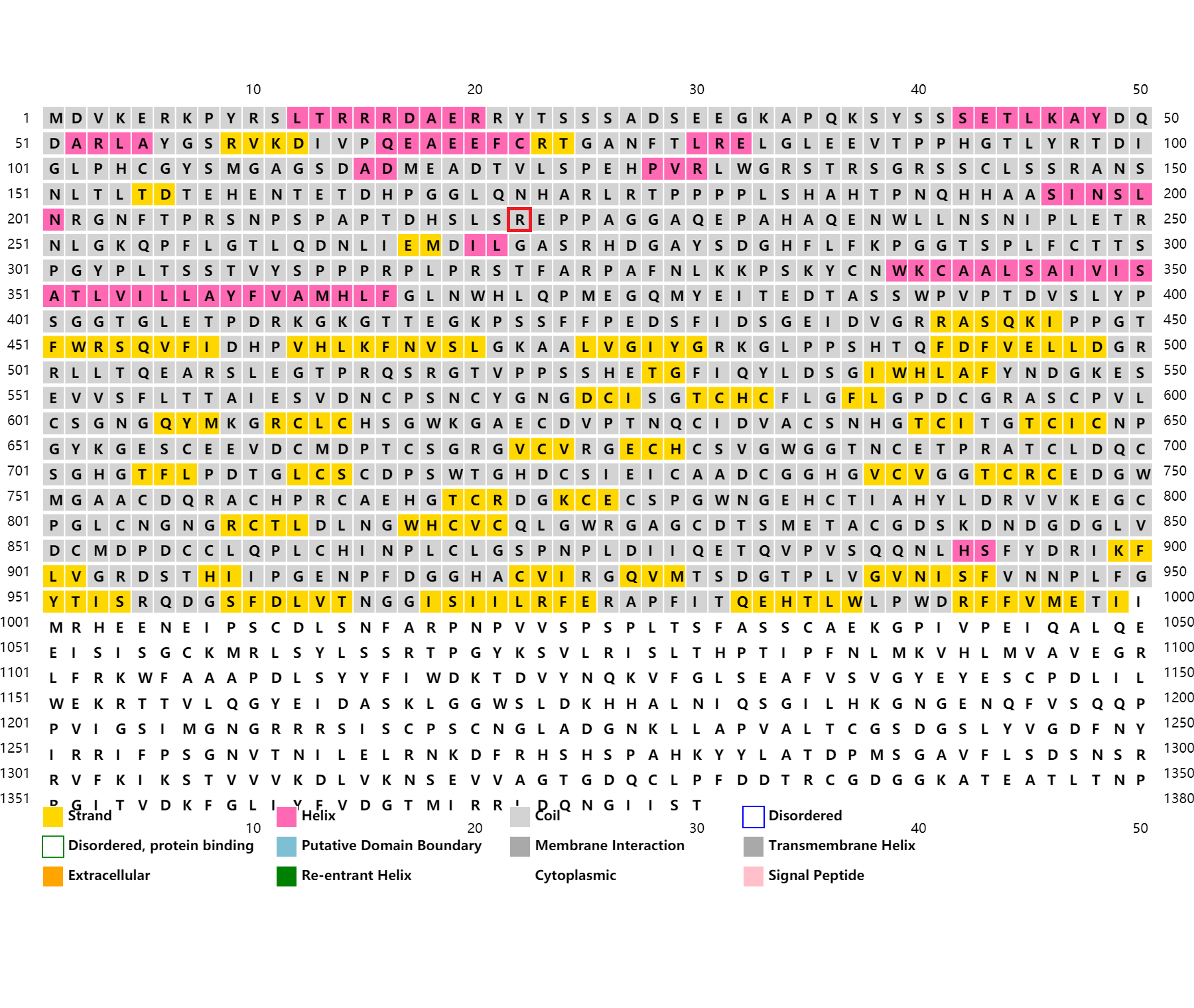

Supplement: Supplementary file 13 [file Image_13.PNG]

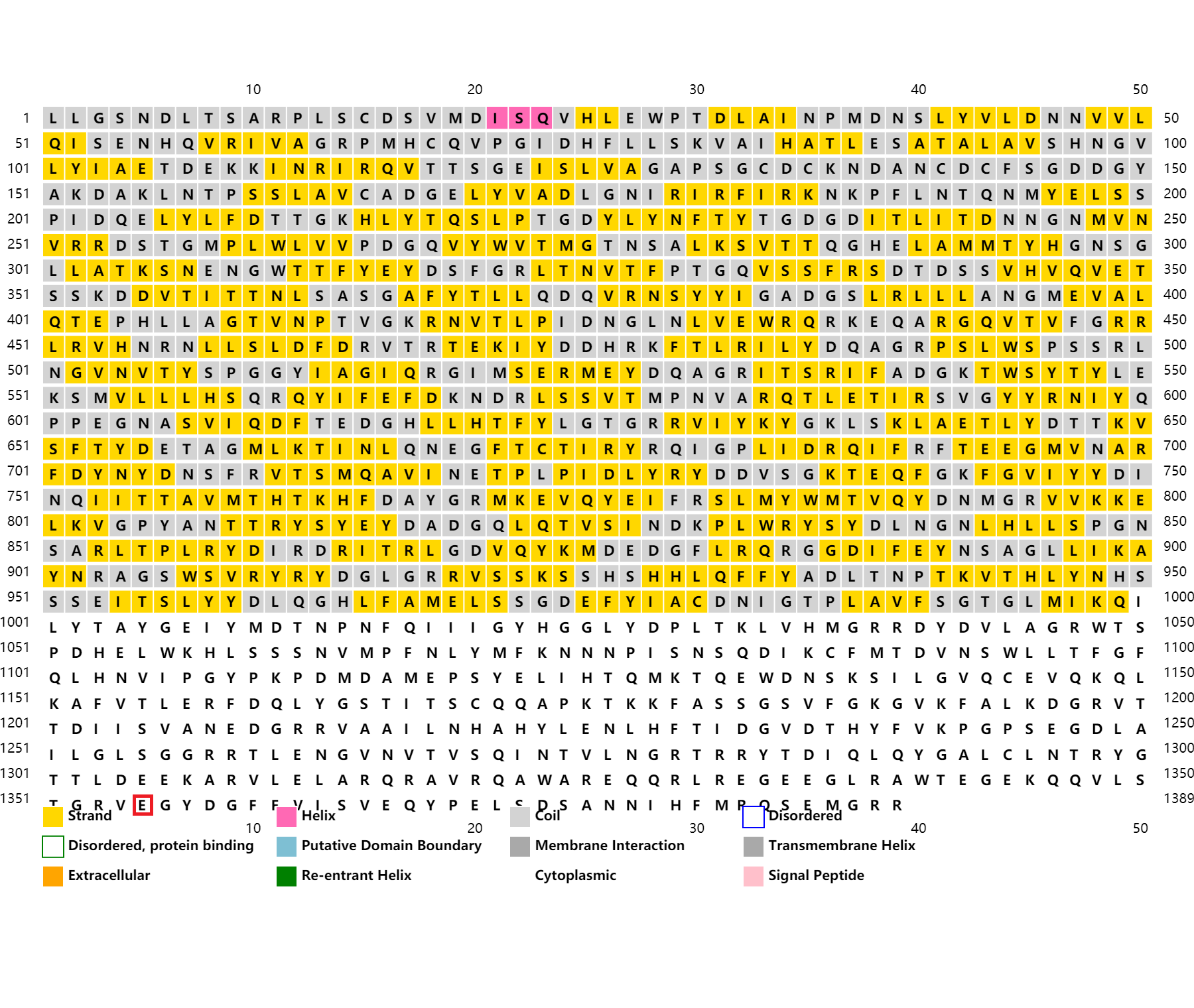

Supplement: Supplementary file 14 [file Image_14.PNG]
